# Microstructure Evolution and Mechanical Properties of Tempered 5140 Alloy Steel after Proton Irradiation at Different Temperatures

**DOI:** 10.3390/ma13132910

**Published:** 2020-06-29

**Authors:** Luanyue Dai, Guangyi Niu, Mingzhen Ma

**Affiliations:** 1 State Key Laboratory of Metastable Materials Science and Technology, Yanshan University, Qinhuangdao 066004, China; dly@rxpopcorn.com (L.D.); jnxyngy@163.com (G.N.); 2 Rema Tip Top (Tianjin) Rubber Technology Co. Ltd., Tianjin 300385, China

**Keywords:** tempered 5140 alloy steel, proton irradiation, microstructure, macroscopic mechanical properties, nanoindentation hardness, hardening mechanism

## Abstract

This article introduces the effect of tempered 5140 alloy steel commonly used in engineering on its structure and mechanical properties under the action of proton irradiation. In the present study, the irradiation energy of 160 keV is applied to experimentally investigate the proton irradiation with different cumulative fluences on the tempered 5140 alloy steel. The effect of the cumulative fluence of the proton irradiation on the microstructure evolution of tempered 5140 alloy steel is studied through transmission electron microscopy. Moreover, the morphology of the tensile fracture is analyzed by scanning electron microscope. The effect of the cumulative fluence of the proton irradiation on the nanomechanical properties of tempered 5140 alloy steel is investigated with a nanomechanical tester. It is found that the surface hardening effect formed by the proton irradiation damage causes the dislocation density in the structure near the tempered 5140 alloy steel surface layer and such effect increases as the proton irradiation cumulative fluence increases. The results obtained show that the yield and tensile strength of the tempered 5140 alloy steel increase slightly as the cumulative fluence of the proton irradiation increases. However, the corresponding elongation decreases. For a stable pressure load of the nanoindentation, the hardness of the nanoindentation of the tempered 5140 alloy steel increases as the proton irradiation fluence increases. However, the corresponding indentation depth decreases. Based on the obtained results, it is concluded that proton irradiation has no significant effect on the macro- and nanomechanical properties of the tempered 5140 alloy steel. This may be attributed to the low energy of the proton irradiation, and the resulting radiation damage only acts on the thin layer of the tempered 5140 alloy steel surface.

## 1. Introduction

Tempered 5140 alloy steel (also called 40Cr alloy) has superior characteristics such as low price, high plasticity, and reasonable workability and weldability so that it is widely applied as a transmission component [1]. Applying different heat treatment systems, this alloy can be utilized to manufacture and process mechanical parts that bear different loads while operating at different speeds, such as knuckles, gears, sprockets, shafts, crankshafts, worms, splined shafts, sliders, collars, pins, and connecting rods [2,3,4,5]. Studies show that 40Cr alloy is one of the most commonly used structural materials for processing and manufacturing machinery in diverse industrial and aerospace applications [6]. Considering the joints in the space manipulator as an example, 40Cr alloy is applied in gear shafts and planetary gears in the planetary gear system, and other moving components, connecting rods, and pins. When the spacecraft tool is in service in an exposed environment outside the cabin, it is often affected by environmental factors, including the alternating temperature, high vacuum, and space particle irradiation. For components and mechanisms installed outside the cabin, it is an enormous challenge to add protective devices, which originates from the need for transfer motions. In fact, it is inevitable for these components to be radiated by various charged particles. Among all charged particles, proton irradiation has a remarkable influence on the organization and performance of materials with relative motion. Consequently, it is of significant importance to investigate these space mechanisms to make a correct evaluation [7]. However, reviewing the literature indicates that no research has been carried out so far to investigate the effect of proton irradiation on the structure and properties of tempered 5140 alloy steel.

A lot of research work has been performed to investigate the effect of proton irradiation on the structure and properties of different alloy steels [8,9,10,11,12]. However, the majority of these studies focused on austenite or ferritic alloy steels, which are commonly used in nuclear reactor pressure vessels that mainly use the proton. The irradiative damage of steel in the nuclear reactor was studied by simulating the neutron irradiation effect. Takeuchi et al. [13] and Zhu et al. [14] showed that the radiation embrittlement of steel in the nuclear reactor pressure vessel (RPV) originates from the tissue changes caused by the irradiation, such as the formation of nanoclusters and the destruction of matrix damage. The matrix damage is considered to be composed of volume defects [15,16] and dislocation loops [13,17,18,19]. Gupta et al. [20] used heavy ions and protons to characterize the microstructure, hardness, deformation, and crack initiation behavior of austenitic stainless steels, and selected iron ions and protons to perform irradiation tests on SA304 stainless steel. It was found that irradiation-induced microstructures consist of dislocation loops and are not affected by the type and dose of irradiated ions. The experimental results showed that the Frank rings of samples irradiated with iron ions and protons were distributed asymmetrically, and most of the rings were distributed between 6 and 24 nm. They demonstrated that the Frank ring number, density, and average size of the samples irradiated with iron ions and protons were basically consistent with the reported values for neutron irradiation and ion irradiation [21,22,23]. They found that when the dose, average number density, and diameter of the Frank ring in the iron ion-irradiated samples increased slightly, the corresponding Frank ring density of the proton-irradiated samples was significantly higher than that of the iron ion-irradiated samples [20].

Although the abovementioned researches are helpful in understanding the organization and performance evolution law of the proton-irradiated metal materials, they were all conducted on nuclear reactor steel. In other words, there is a lack of knowledge about the effect of proton irradiation on the structure and properties of tempered 5140 alloy steel so that research and exploration in this regard is highly demanded. Therefore, it is of great importance from the scientific and engineering point of view to study the action mechanism of proton irradiation on the microstructure evolution and mechanical properties of 5140 alloy steel tempered at different temperatures.

In the present article, we intended to investigate the effects of proton irradiation on the microstructure evolution and macro-mechanical properties of tempered 5140 alloy steel. To this end, proton irradiation experiments with different cumulative fluences were conducted on the tempered 5140 alloy steel. Moreover, the correlation between the elastic modulus of tempered 5140 alloy steel, the hardness of nanoindentation, and the depth of nanoindentation were analyzed with a nanoindentation mechanical tester. It is expected that the present article can provide useful information for evaluating the effect of proton irradiation on the microstructure evolution and properties of tempered 5140 alloy steel.

## 2. Materials and Methods

### 2.1. Experimental Materials and Heat Treatment Process

In the present study, the experiment was conducted on a round bar made of tempered 5140 alloy steel (the manufacturer was Beijing Shougang Co., Ltd., Beijing, China). The diameter and density of the bar were D = 20mm and 7.77g/cm^3^, respectively. Table 1 shows the composition of the 5140 alloy.

The heat treatment of the tempered 5140 alloy steel was formulated in accordance with the national standard GB/T 3077-1999 [24]. The heat treatment process was performed as follows: firstly, the specimen made from the 5140 alloy steel was heated to 850 °C for 1 h at a heating rate of 10 °C/min. Secondly, the specimen was put into cooling oil for quenching after being released. Finally, it was tempered at 480 °C, 520 °C, and 560 °C, respectively. The heat treatment test device was a vacuum tube heat treatment furnace (SK-G06143, Tianjin Zhonghuan Electric Furnace Co., Ltd., Tianjin, China). The heat treatment furnace was heated under the protection of argon gas. The maximum temperature, heating rate, and the temperature accuracy were 1300 ± 1 °C, 10 ± 1 °C/min, respectively.

### 2.2. Proton Irradiation Test

**Figure 1:**
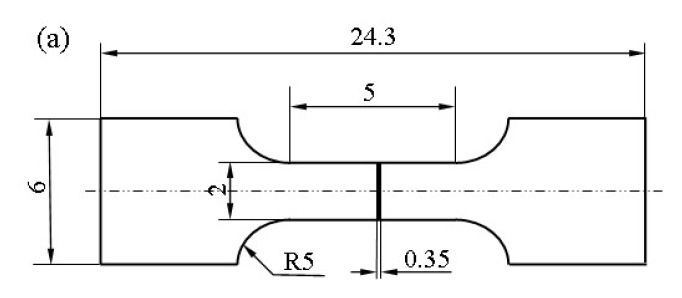

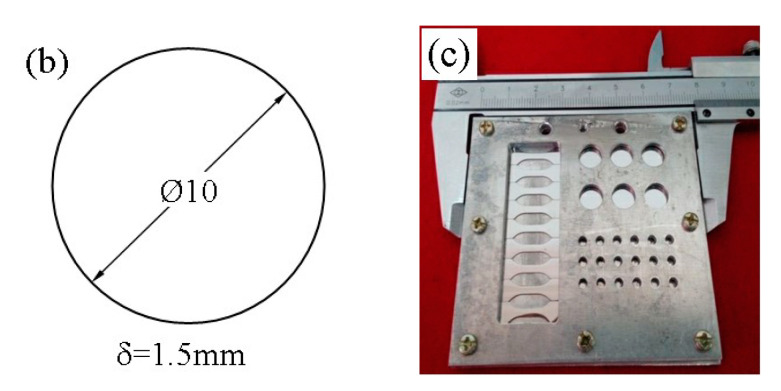
Dimensions of irradiated specimens of tempered 5140 alloy steel. (**a**) Micro tensile specimen. (**b**) Nanoindentation specimen. (**c**) Clamping die of proton irradiated sample.

The proton irradiation test was carried out on a positive ion irradiation simulator at the Harbin Institute of Technology (Non standard equipment, B. Verkin Institute of Low Temperature Physics & Engineering, Kharkov, Ukraine). Prior to the proton irradiation test, the tempered 5140 alloy steel was processed into sheet-shaped tensile specimens and disc-shaped specimens, as shown in Figure 1a,b, respectively. Then, the surface was cleaned and the alloy was installed into the irradiation sample clamping mold, as shown in Figure 1c. Then, the mold was placed on the workbench in the positive ion irradiation simulation instrument. It is worth noting that during the proton irradiation test, the exposed surface of the sample was perpendicular to the proton beam generator. Meanwhile, proton irradiation experimental conditions were as follows: the irradiation vacuum environment and the irradiation energy were 10^−3^~10^−4^ Pa and 160 keV, respectively. The irradiation flux and temperature were 1.25 × 10^11^ protons/cm^2^·s and room temperature, respectively. The cumulative fluences of the irradiation were 2 × 10^14^ protons/cm^2^, 2 × 10^15^ protons/cm^2^, and 2 × 10^16^ protons/cm^2^. It should be noted that a proton is a charged particle that can penetrate the matter depending on its energy. High-energy particles can change the physical properties of the matter, while low-energy particles can only change the surface properties of the matter. Considering the capacity limitations of the test equipment, the 160 keV irradiation energy used in the irradiation test in this study is in the low energy category.

### 2.3. Simulation of Proton Irradiation Damage

The evaluation of proton irradiation damage was simulated by Stopping and Range Ions Matter (SRIM) software (SRIM-2013-Pro.e, International Business Machines Co., Ltd., Armonk, NY, USA). The mass ratio of 5140 alloy steel constituent elements was converted into atomic ratio in the SRIM simulation program, and the irradiation energy was calculated to be 160 keV in the simulation results of time proton transport and distribution in 5140 alloy steel.

### 2.4. XRD (X-Ray Diffraction) Analysis

X-ray diffraction (XRD) analysis was carried out on a D/max-2500/PC type X-ray diffraction instrument (the manufacturer was Japan Rigaku Co., Ltd., Tokyo, Japan). During the experiment, the proton irradiation surface of the sample was taken using a Cu target, Kα rays, a characteristic wavelength of 1.540598 Ǻ, a scanning range of 20–110°, and a step length of 0.02°. The tested working voltage was 40 kV, and the working current was 100 mA.

### 2.5. Mechanical Properties Test

#### 2.5.1. Tensile Test of the Irradiated Specimen

Using the INSTRON 5948 mechanical property test system (the manufacturer was INSTRON Co., Ltd., Boston, MA, USA), the tensile strength test of the tempered 5140 alloy steel before and after the irradiation was conducted. It should be noted that the tensile strain rate during the test was set to 1 × 10^−3^s^−1^. The temperature of the tensile test was room temperature in accordance with the national standard GB/T 228-2002 [25].

#### 2.5.2. Nanoindentation Hardness Test of Irradiated Samples

The disc-shaped sample after the proton irradiation was subjected to a nanoindentation test in a nanoindentation mechanical tester using a Berkovich indenter (the manufacturer was Hysitron Co., Ltd., Minneapolis, MN, USA). Three loads were applied during the test. The applied loads were 4000 µN, 6000 µN, and 8000 µN, respectively. Moreover, the pressure was maintained for 1 min.

### 2.6. TEM (Transmission Electron Microscope) and SEM (Scanning Electron Microscope) Analysis

The TEM (transmission electron microscope) sample was prepared by a single-sided thinning method from the unirradiated side (i.e., backside). The irradiated sample was grounded from the backside to a thickness of about 30 μm with metallographic sandpaper. Then, it was punched into a disc with a diameter of 3 mm. Finally, single-sided ion thinning perforation was performed to obtain a TEM sample. Finally, the microstructure analysis was carried out using a transmission electron microscope, model JEOL2010 (manufacturer, Japan Electronics Co., Ltd., Tokyo, Japan), while the acceleration voltage was set to 200 kV, and the emission current was 50 pA.

The tensile fracture analysis of tempered 5140 alloy steel was performed using a high-resolution field emission scanning electron microscope (SEM), model S-4800 (manufacturer, Hitachi Co., Ltd., Tokyo, Japan). In order to avoid contamination and damage of the tensile fracture, the fracture was covered with a clean test paper during the sampling process, and then it was placed in a scanning electron microscope to observe and analyze the tensile fracture. The tested acceleration voltage was 15 kV and an emission current is 10 µA.

## 3. Results

### 3.1. XRD

**Figure 2:**
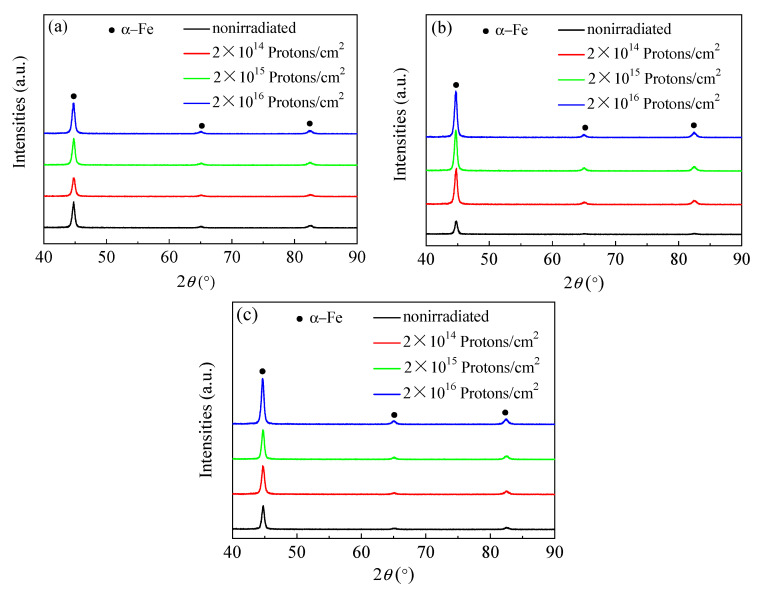
XRD diffraction patterns of 5140 steel irradiated by proton at different tempering temperatures: (**a**) 480 °C tempering, (**b**) 520 °C tempering, (**c**) 560 °C tempering.

Figure 2 is the cumulative injection of 5140 alloy steel after quenching, after tempering at 480 °C, 520 °C, and 560 °C at 2 × 10^14^ protons/cm^2^, 2 × 10^15^ protons/cm^2^, and 2 × 10^16^ protons/cm^2^ proton irradiation, respectively, and XRD diffraction patterns under the same conditions as before and without proton irradiation. As can be seen from Figure 2, only three α-Fe phases were observed in the XRD diffraction patterns of the proton irradiation experiments under three different tempering temperatures. The existence of diffraction peaks of other phases was not found. This may be because the energy of 160 keV proton irradiation was a lower energy, which was not enough to induce the formation of new phases.

### 3.2. Simulation Results of Proton Irradiation Damage

**Figure 3:**
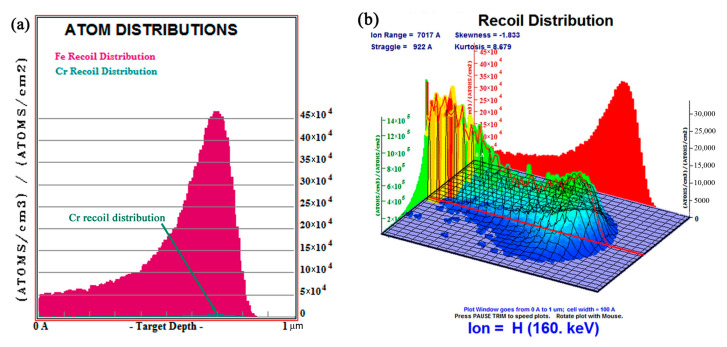
Distribution of the transport and damage position of H^+^ protons on the surface layer of tempered 5140 alloy steel: (**a**) distribution of Fe and Cr recoil atoms along the depth of incidence, (**b**) three-dimensional distribution of vacancies formed by Fe, Cr recoil atoms, and H^+^ proton irradiation along the depth of incidence.

When analyzing the action mechanism of the proton irradiation on the microstructure evolution of the tempered 5140 alloy steel, the transport of ions in matter (TRIM) in stopping and range ions matter (SRIM) simulation software was used according to the proton irradiation test parameters of the present study. Figure 3 shows that the ion transport simulation calculation module calculated the distribution of the transport and damage position of the cumulative fluence of 2 × 10^16^ protons/cm^2^ H^+^ protons on the surface layer of the irradiated sample.

Figure 3a shows the depth distribution of the Fe and Cr recoil atoms formed by the irradiation of H^+^ protons in the matrix near the tempered 5140 alloy steel surface layer along the incident direction. Figure 3a shows that the dark red skewed distribution pattern is the depth distribution of Fe atoms along the incident direction, and its maximum peak position was about 0.7 µm. The dark blue distribution pattern is the distribution of Cr atoms along the incident direction. Due to the small Cr content of the tempered 5140 alloy steel, the distribution pattern is almost close to the bottom abscissa, which is difficult to identify. In order to facilitate the viewing of the peak position of the Cr atom distribution, the corresponding position is marked with a dark blue arrow in the Figure 3a. Moreover, the maximum peak position of Cr atoms was about 0.7 µm from the surface.

Figure 3b shows the three-dimensional distribution diagram of vacancies formed by the irradiation of Fe and Cr recoil atoms and H^+^ protons along the depth of the incidence. It was observed that a large number of vacancies (grid diagram composed of yellow, green and blue colors) appeared near the tempered 5140 alloy steel sample surface layer.

### 3.3. Microstructure Evolution of the Tempered 5140 Alloy Steel before and after Proton Irradiation

**Figure 4:**
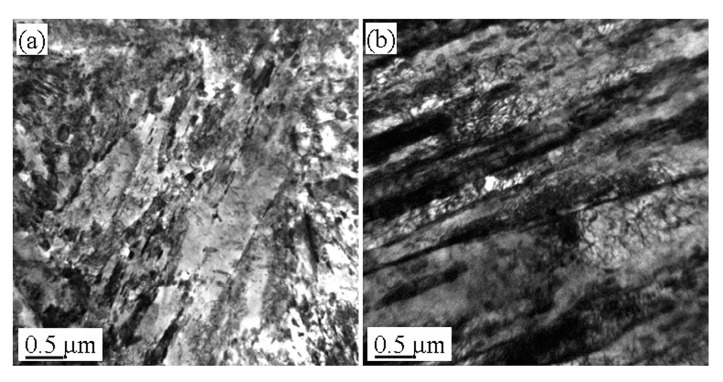

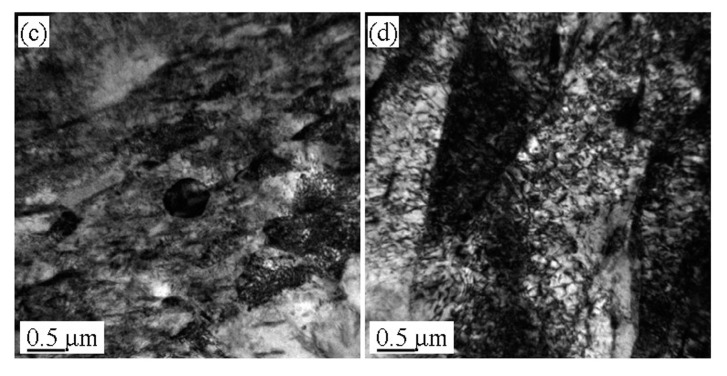
Microstructure of tempered 5140 steel tempered at 480 °C before and after different accumulated proton irradiation observed by TEM pictures of (**a**) 0 protons/cm^2^, (**b**) 2 × 10^14^ protons/cm^2^, (**c**) 2 × 10^15^ protons/cm, (**d**) 2 × 10^16^ protons/cm^2^.

**Figure 5:**
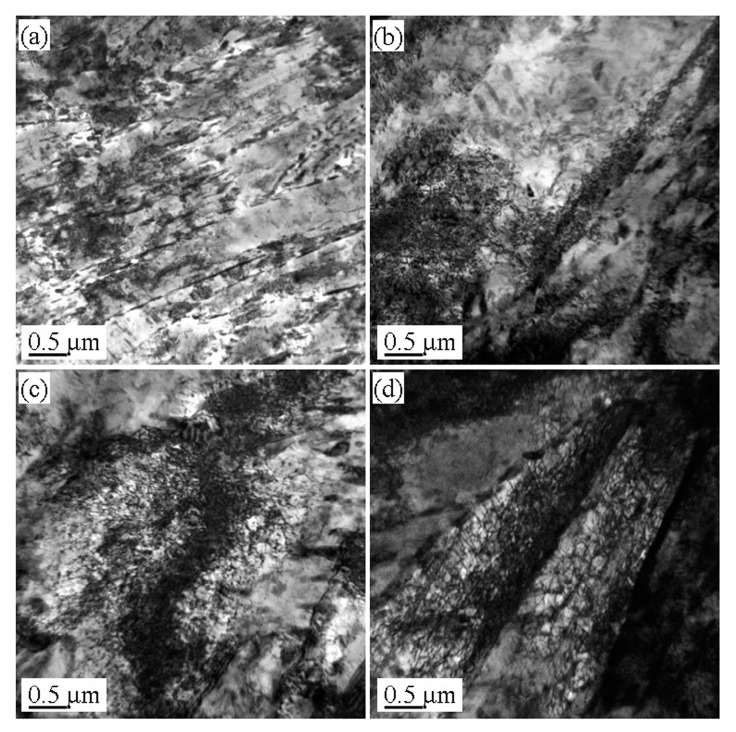
Microstructure of tempered 5140 steel tempered at 520 °C before and after different accumulated proton irradiation observed by TEM pictures of (**a**) 0 protons/cm^2^, (**b**) 2 × 10^14^ protons/cm^2^, (**c**) 2 × 10^15^ protons/cm^2^, (**d**) 2 × 10^16^ protons/cm^2^.

**Figure 6:**
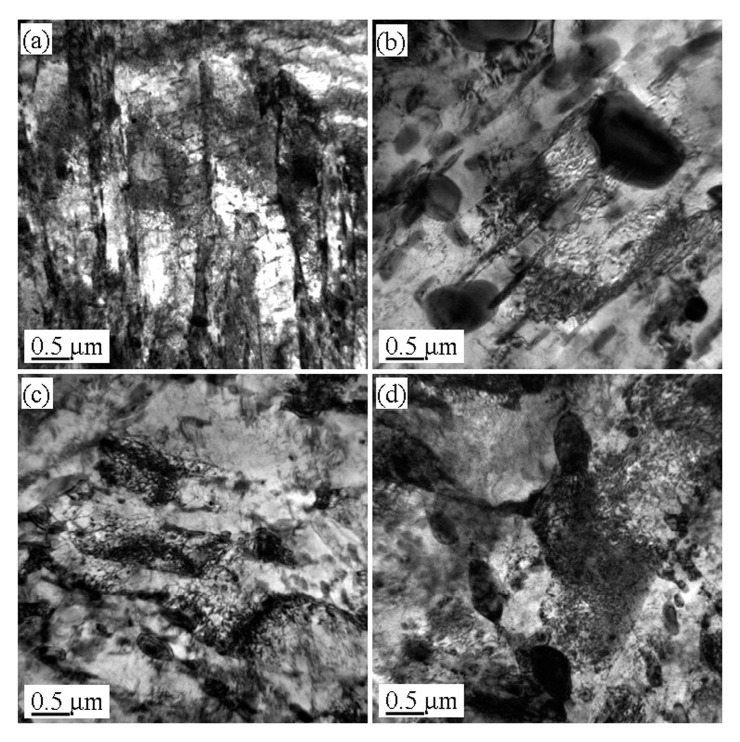
Microstructure of tempered 5140 steel tempered at 560 °C before and after different accumulated proton irradiation observed by TEM pictures of (**a**) 0 protons/cm^2^, (**b**) 2 × 10^14^ protons/cm^2^, (**c**) 2 × 10^15^ protons/cm^2^, (**d**) 2 × 10^16^ protons/cm^2^.

Figure 4, Figure 5 and Figure 6 show the microstructures in the transmission electron microscope images of the 5140 alloy steel after tempering at 480 °C, 520 °C, and 560 °C after different proton irradiation evolutions, respectively.

Figure 4a shows that the structure of the tempered steel at 480 °C was fine lath martensite. Meanwhile, black precipitates were also observed. Figure 4b–d indicate that as the cumulative fluence of the proton irradiation increased, the dislocation density of the sample also increased, and the configuration of dislocations was determined by the dislocations. This may be because as the cumulative fluence of proton irradiation increased, the degree of lattice distortion of the sample surface caused by the irradiation damage increased, resulting in an increase in dislocation density.

It should be indicated that the loop was composed of dislocation lines intertwined with each other. Figure 5 illustrates the microstructure of the 5140 alloy steel tempered at 520 °C after the proton irradiation. It was observed that the sample without proton irradiation (Figure 5a) and the sample with proton irradiation (Figure 5b–d) were similar to that in Figure 4. However, the size of the martensite lath increased, and the dislocation density decreased. Figure 6 shows that when the tempering temperature was set to 560 °C, the martensite lath of the tempered 5140 alloy steel grew further, and the dislocation density in its microstructure decreased. This was mainly attributed to the increment in the tempering temperature.

### 3.4. Macro Mechanical Properties of the Tempered 5140 Alloy Steel before and after Irradiation with Protons

**Figure 7:**
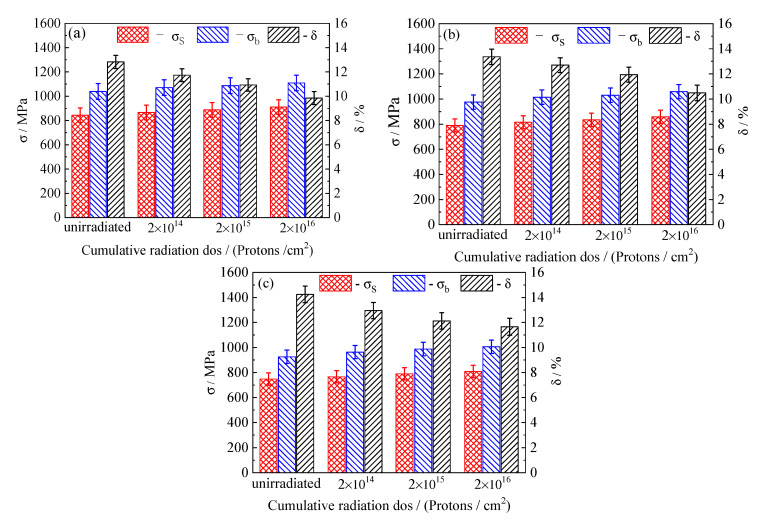
Mechanical properties of different tempered 5140 alloy steel before and after proton irradiation: (**a**) tempering at 480 °C, (**b**) tempering at 520 °C, and (**c**) tempering at 560 °C.

Figure 7a shows that the yield strength, tensile strength, and elongation of the 5140 alloy steel tempered at 480 °C without proton irradiation are 843 MPa, 1039 MPa, and 12.8%, respectively. After the cumulative fluence the yield strength, tensile strength, and elongation after irradiation of protons of 2 × 10^14^ protons/cm^2^, 2 × 10^15^ protons/cm^2^, and 2 × 10^16^ protons/cm^2^ are 865 MPa, 1071 MPa, and 11.7%; 887 MPa, 1086 MPa, and 10.9% vs. 910 MPa, 1109 MPa, and 9.8%, respectively. It is found that the yield strength of the tempered 5140 alloy steel after the proton irradiation in comparison with those without the proton irradiation increased by 2.6%, 5.2%, and 7.9%, respectively. Figure 7b shows that for the 5140 alloy steel tempered at 520 °C, the yield strength of the 5140 alloy steel irradiated with protons increased by 3.1%, 5.7%, and 8.7% compared with those without proton irradiation. Moreover, Figure 7c shows that when the tempering temperature increased to 560 °C, the yield strength of the 5140 alloy steel increased as the cumulative fluence of the proton irradiation increased. Compared with non-irradiated samples, the increments in the yield strength were 2.4%, 5.6%, and 8.0%, respectively.

Through the analysis of the mechanical performance data shown in Figure 7, it was observed that when the proton irradiation cumulative fluence was unchanged, the strength of the tempered 5140 alloy steel decreased as the tempering temperature increased. For a specific tempering temperature, the yield and tensile strengths of the tempered 5140 alloy steel increased after proton irradiation. It was found that the proton irradiation cumulative fluence had a negative impact on the elongation. As the cumulative fluence of proton irradiation increased, the elongation decreased. From the above experimental results, it was concluded that although the proton irradiation increased the yield and tensile strengths of tempered 5140 alloy steel, such increment was very small, varying only in the range of 2.4% to 8.7%. 

### 3.5. Tempering Fracture Morphology of 5140 Alloy Steel before and after Irradiation with Protons

**Figure 8:**
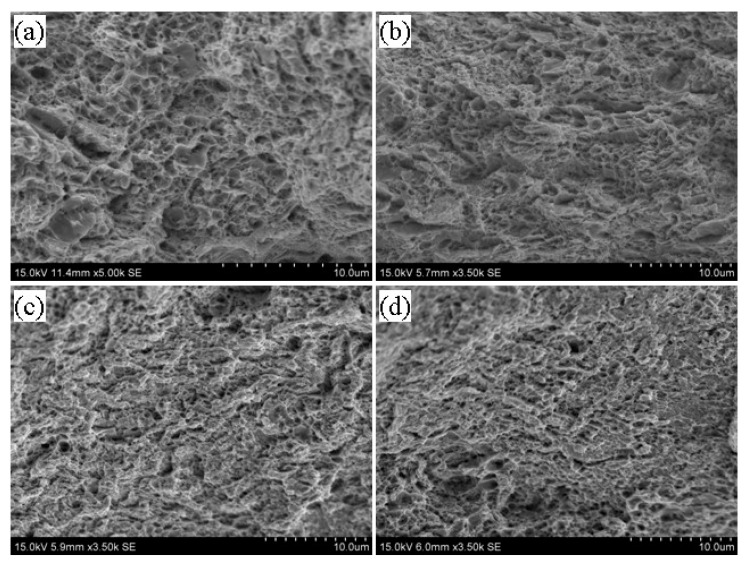
Fracture morphology of tempered 5140 alloy steel before and after tempering at 480 °C (**a**) 0 Protons/cm^2^ (**b**) 2 × 10^14^ Protons/cm^2^ (**c**) 2 × 10^15^ Protons/cm^2^ (**d**) 2 × 10^16^ Protons/cm^2^.

**Figure 9:**
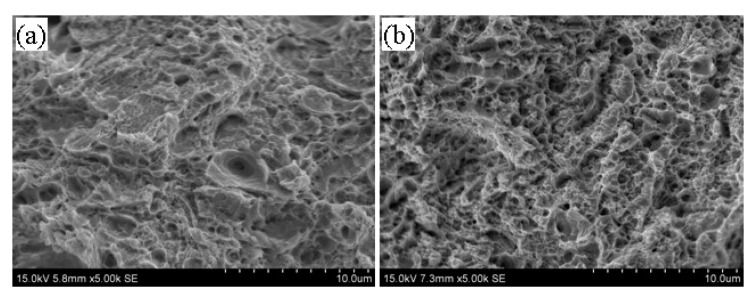

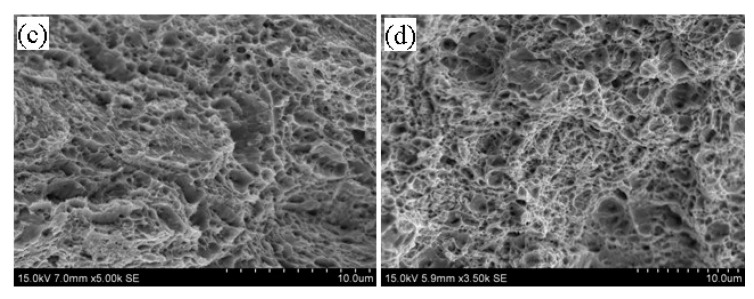
Fracture morphology of tempered 5140 alloy steel before and after tempering at 520 °C: (**a**) 0 protons/cm^2^, (**b**) 2 × 10^14^ protons/cm^2^, (**c**) 2 × 10^15^ protons/cm^2^, (**d**) 2 × 10^16^ protons/cm^2^.

**Figure 10:**
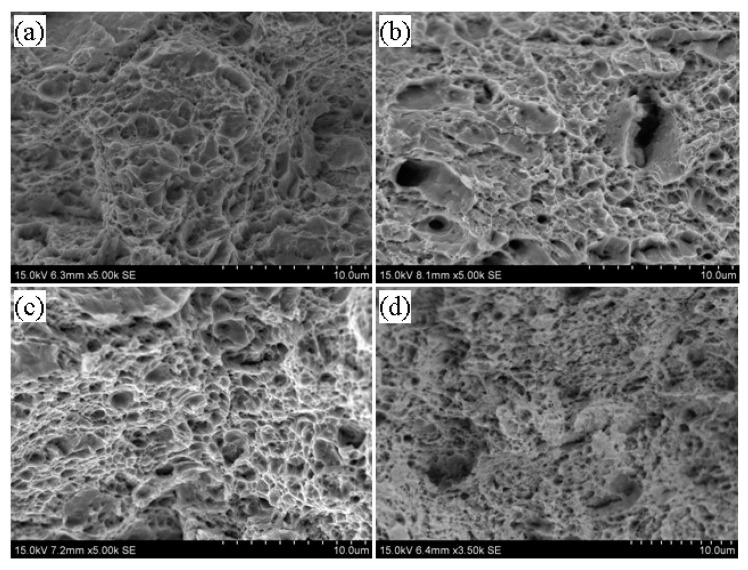
Fracture morphology of tempered 5140 alloy steel before and after tempering at 560 °C (**a**) 0 Protons/cm^2^ (**b**) 2 × 10^14^ Protons/cm^2^ (**c**) 2 × 10^15^ Protons/cm^2^ (**d**) 2 × 10^16^ Protons/cm^2^.

Figure 8, Figure 9 and Figure 10 illustrate the fracture morphology of the tempered 5140 alloy steel before and after irradiation with different cumulative fluence protons. The tensile fracture of the tempered 5140 alloy steel shown in the figure was sampled near the proton irradiated surface. Figure 8 shows the tensile fracture morphology of 5140 alloy tempered at 480 °C before and after proton irradiation. Figure 8 indicates that although the fracture morphology of tempered 5140 alloy steel before and after the proton irradiation was composed of tough dimples, the size of fracture dimples without the proton irradiation was relatively large. Meanwhile, the deformation of the tough tear edge was obvious. It was observed that as the cumulative fluence of proton irradiation increased, the size of the dimple decreased, and the tearing edge and plastic deformation of the dimple edge decreased too.

Figure 9 presents the tensile fracture morphology of 5140 alloy steel tempered at 520 °C before and after proton irradiation. It was observed that the fracture morphology at this time was very similar to the fracture morphology during the tempering at 480 °C. However, the dimple size and the plasticity of the tearing edge of the dimple was larger than that of tempering at 480 °C. Moreover, the deformation degree increased due to the increase in the tempering temperature.

Figure 10 illustrates the tensile fracture morphology of 5140 alloy steel tempered at 560 °C before and after the proton irradiation. It was observed that the fractures at this time had similar morphological features as those in Figure 8 and Figure 9, and the fractures that were not irradiated with protons showed relatively large dimples. Figure 10 indicates that as the cumulative fluence of the proton irradiation increased, the size of the dimples with fracture decreased. However, its fracture type still showed a ductile fracture.

Based on the analysis of the fracture morphology shown in Figure 8, Figure 9 and Figure 10, it was found that for a constant tempering temperature, the tough dimples of the tempered 5140 alloy steel fracture became smaller as the cumulative fluence of proton irradiation increased. On the other hand, the deformation of the dimple and the tearing edge of the edge also decreased.

When the cumulative fluence of the irradiation was unchanged, the dimple of the tempered 5140 alloy steel fracture increased as the tempering temperature increased. Since the fracture of the tensile specimen was actually near the surface of the surface irradiated by protons, it would have a certain influence on the fracture morphology and the size of the dimples of the tensile specimen. That is, as the cumulative fluence of proton irradiation increased, the hardening tendency of the irradiated surface also increased, so it showed that the size of the dimple decreased and the degree of deformation of the edge of the dimple decreased. However, the change of fracture morphology was basically consistent with the change trend of tensile strength, and it still showed ductile fracture. The fracture type showed a ductile fracture.

### 3.6. Tempered Nanomechanical Properties of 5140 Alloy Steel before and after Proton Irradiation

#### 3.6.1. Nanoindentation Hardness and Depth Curves of the Tempered 5140 Alloy Steel before and after Irradiation with Protons

**Figure 11:**
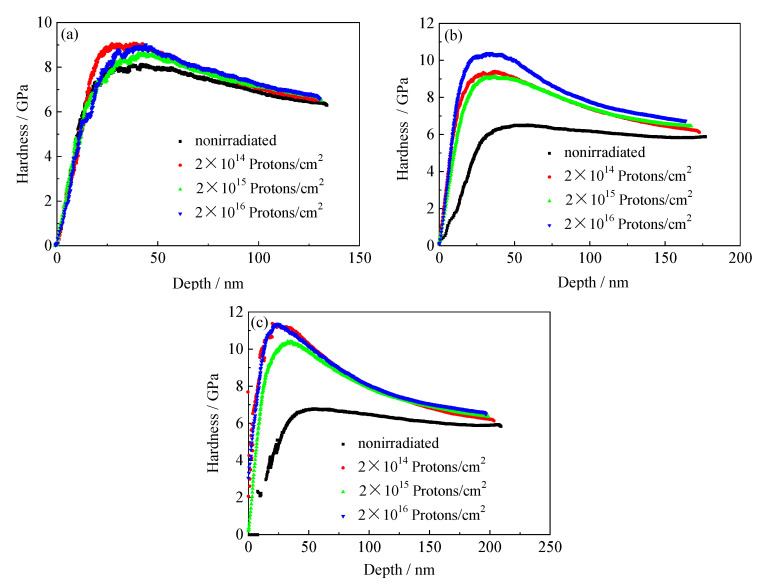
Variation of indentation hardness of tempered 5140 alloy steel tempered at 480 °C with depth under different loads before and after proton irradiation: (**a**) 4000 μN, (**b**) 6000 μN, (**c**) 8000 μN.

**Figure 12:**
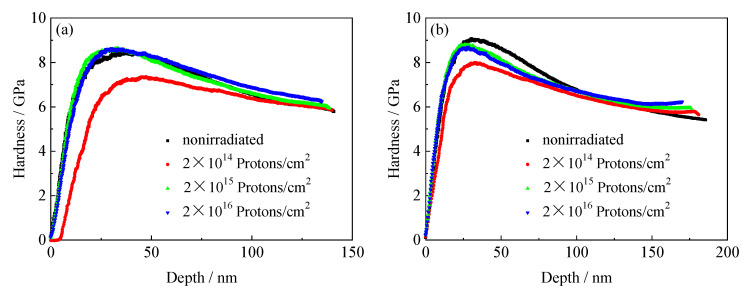

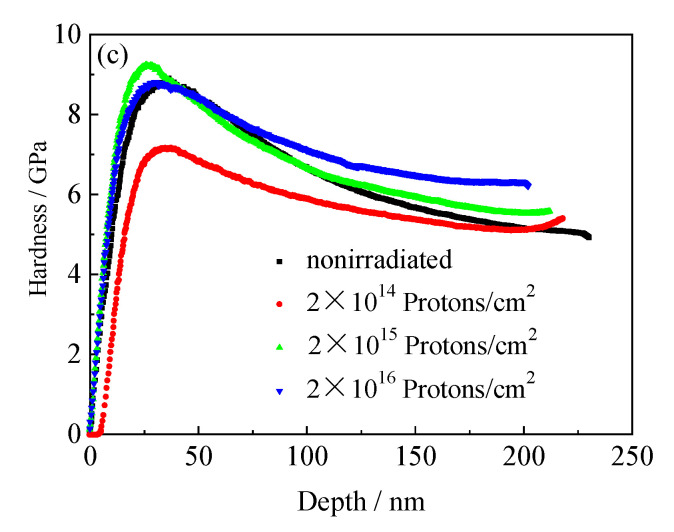
Variation of indentation hardness of tempered 5140 alloy steel tempered at 520 °C with depth under different loads before and after proton irradiation: (**a**) 4000 μN, (**b**) 6000 μN, (**c**) 8000 μN.

**Figure 13:**
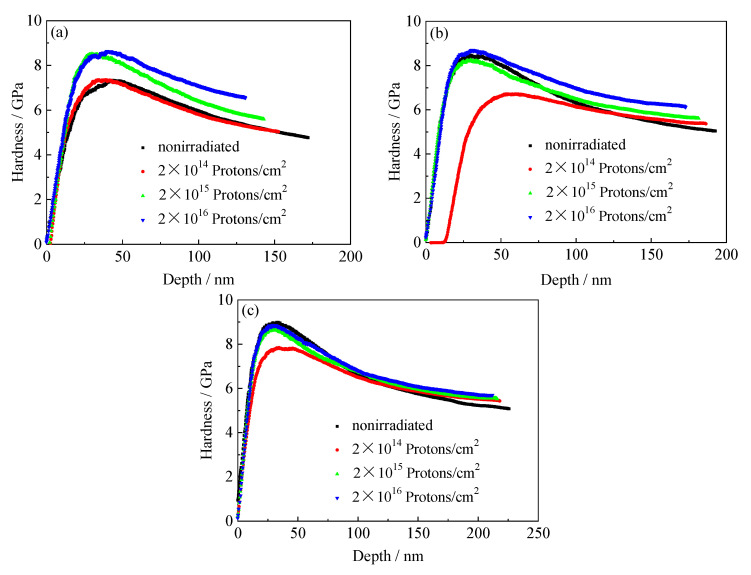
Variation of indentation hardness of tempered 5140 alloy steel tempered at 560 °C with depth under different loads before and after proton irradiation: (**a**) 4000 μN, (**b**) 6000 μN, (**c**) 8000 μN.

Figure 11, Figure 12 and Figure 13 show the curves of the nanoindentation hardness and nanoindentation depth of the tempered 5140 alloy steel under different pressure loads as the cumulative fluence of the proton irradiation increased. It was observed that the nanoindentation hardness and nanoindentation depth of the tempered 5140 alloy steel were closely correlated to the three experimental parameters of the tempering temperature, cumulative fluence of the proton irradiation, and pressure load. When the tempering temperature was constant, the hardness of the nanoindentation increased as the cumulative fluence of the proton irradiation increased, while it decreased as the pressure load increased. The depth of the nanoindentation decreased as the cumulative fluence of the proton irradiation increased. When the cumulative fluence of the proton irradiation was constant, the hardness of the nanoindentation decreased as the tempering temperature and load increased. Moreover, the depth of the nanoindentation increased as the tempering temperature and load increased. Furthermore, when the pressure load was constant, the hardness of the nanoindentation increased as the cumulative fluence of the proton irradiation increased, while it decreased as the tempering temperature increased.

#### 3.6.2. Nanomechanical Properties of the Tempered 5140 Alloy Steel before and after Irradiation with Protons

Table 2 shows the nanomechanical properties of the tempered 5140 alloy steel at different temperatures before and after proton irradiation.

It was found that the nanoindentation elastic modulus of the tempered 5140 alloy steel underwent proton irradiation with cumulative fluences of 2 × 10^14^ protons/cm^2^, 2 × 10^15^ protons/cm^2^, and 2 × 10^16^ protons/cm^2^, respectively. It was observed that before and after proton irradiation, no significant changes occurred. However, when the effects of the three influencing factors of the tempering temperature, pressure load, and cumulative fluence of the proton irradiation were analyzed, some slight changes were observed. When the tempering temperature was unchanged, the elastic modulus had a slight downward trend as the pressure load increased. Moreover, the elastic modulus increased slightly as the cumulative fluence of the proton irradiation increased. When the pressure load was constant, the elastic modulus decreased as the tempering temperature increased. Moreover, the elastic modulus changed irregularly as the cumulative fluence of the proton irradiation increased. However, the overall change results showed an increasing trend. When the cumulative fluence of the proton irradiation was unchanged, the elastic modulus decreased slightly as the temperature and load increased. The abovementioned analysis shows that the effect of the cumulative fluence of the proton irradiation on the elastic modulus of the tempered 5140 alloy steel was lower than the effect of load and temperature among the three influencing factors of tempering temperature, pressure load, and cumulative fluence of proton irradiation. The range of change under load was about ± 3–5 GPa (about ± 1.5–2.5%), while the range of change under the effect of the tempering temperature and cumulative fluence of the proton irradiation was about ± 1–2 GPa (about ± 0.5–1%). It should be noted that the elastic modulus, as a physical property of the tempered 5140 alloy steel, reflects an important performance index of its ability to resist the elastic deformation under external force. From a macro perspective, the elastic modulus is a measurement of the ability of an object to resist the elastic deformation. From a micro perspective, it is a reflection of the bonding strength between atoms, ions, or molecules.

The effect of the proton irradiation on the hardness of the tempered 5140 alloy steel nanoindentation could be analyzed by the hardness test data in Table 2. The tempered 5140 alloy steel underwent cumulative fluences of 2 × 10^14^ protons/cm^2^, 2 × 10^15^ protons/cm^2^, and 2 × 10^16^ protons/cm^2^, before and after the proton irradiation. When the tempering temperature was unchanged, the nanoindentation hardness of the tempered 5140 alloy steel increased as the cumulative fluence of load and proton irradiation increased. Moreover, when the load was constant, the hardness of the nanoindentation increased as the proton irradiation cumulative fluence increased, while it decreased as the tempering temperature increased. When the cumulative fluence of the proton irradiation was unchanged, the nanoindentation hardness changed little as the pressure load increased. However, as the tempering temperature increased, the nanoindentation hardness decreased significantly. Taking the change of the nanoindentation hardness of the 5140 alloy steel tempered at 480 °C as an example, after the cumulative fluences of 2 × 10^14^ protons/cm^2^, 2 × 10^15^ protons/cm^2^, and 2 × 10^16^ protons/cm^2^, when the pressure load was 4000 μN, the corresponding increment of the nanoindentation hardness was 3.97%, 4.29%, and 4.61%, respectively. Moreover, when the pressure load was 6000 μN, the corresponding increment range of the nanoindentation hardness was 4.26%, 9.88%, and 14.48%, respectively. Furthermore, when the pressure load was 8000 μN, the increase in the nanoindentation hardness was 4.43%, 8.52%, and 10.9%, respectively.

The hardness of the nanoindentation of 5140 alloy steel tempered at 520 °C and 560 °C after different cumulative fluences proton irradiation also showed the same trend. It was observed that the nanoindentation hardness of tempered 5140 alloy steel increased monotonously as the cumulative fluence of the proton irradiation increased. Table 2 shows that the increase in the hardness of the nanoindentation caused by the irradiation hardening and proton irradiation was mostly less than 15%. Moreover, the highest increase in the hardness of the nanoindentation was 37%, which was basically consistent with the variation of macroscopic mechanical properties.

## 4. Discussion

### 4.1. Microstructure Evolution Mechanism of the Tempered 5140 Alloy Steel after the Proton Irradiation

Proton irradiation causes changes in the physical and mechanical properties of the material. Moreover, it changes the organization and structure of the material. The defects produced in the crystalline materials after the proton irradiation are generally called the irradiation damage. For metallic materials, the simplest irradiation defect is an isolated point defect, which is usually composed of a dislocated atom into a gap atom and the lattice vacancy after dislocation, which is also known as the Frenkel defect pair [26]. Defects caused by the radiation damage often lead to changes in the microstructure of the material.

The microstructural changes caused by the proton irradiation are generally considered to be caused by solute, especially copper-rich steel, and matrix damage [18,27]. Figure 3a shows that the radiation damage locations calculated by the simulation are consistent with the simulation results calculated in the study of Hosemann et al. and Wan et al. [28,29] and the experimental test results in the studies of Nix et al. [30] and Janni [31]. It can be seen from Figure 3a that the incident H^+^ protons collide elastically and inelastically with the atoms in the tempered 5140 alloy steel sample, thereby transferring energy to the tempered 5140 alloy steel substrate through the irradiated surface. When the matrix atoms, also called recoil atoms, are hit by H^+^ protons and receive enough energy, they will escape from the lattice and escape into interstitial atoms. Therefore, a large number of dislocation rings or dislocation clusters are formed near the surface layer of the tempered 5140 alloy steel.

The abovementioned analysis shows that protons have the following two effects on the irradiation of the tempered 5140 alloy steel: (1) a transfer of energy to the tempered 5140 alloy steel sample; and (2) dislocation of the atoms in the irradiated sample and the generation of points defect. Both of these effects can rearrange the atoms near the surface of the tempered 5140 alloy steel sample and change the dislocation configuration in its microstructure. Figure 4, Figure 5 and Figure 6 demonstrate the evolution of this microstructure.

**Figure 14:**
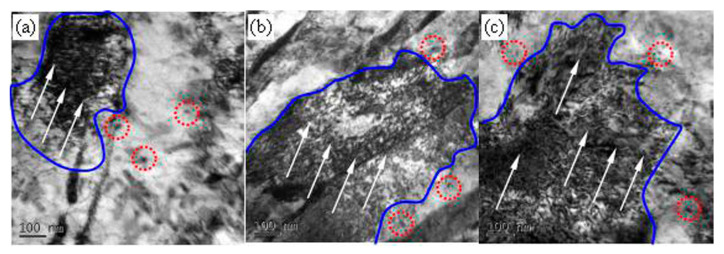
The induced radiation observed by high-power TEM images when the cumulative fluences of the 5140 alloy steel at 480 °C were irradiated with 2 × 10^14^ protons/cm^2^ (**a**), 2 × 10^15^ protons/cm^2^ (**b**), and 2 × 10^16^ protons/cm^2^. (**c**) Changes in the density of dislocation loops and dislocation clusters in the microstructure caused by the damage.

Figure 14 shows the induced radiation observed by high-power TEM images when the cumulative fluences of the 5140 alloy steel at 480 °C were irradiated with 2 × 10^14^ protons/cm^2^, 2 × 10^15^ protons/cm^2^, and 2 × 10^16^ protons/cm^2^ changes in the density of dislocation loops and dislocation clusters in the microstructure caused by the damage.

The high-magnification TEM image illustrated in Figure 14 shows that the microstructure near the surface layer of the 5140 alloy steel after tempering and proton irradiation is composed of a large number of intertwined dislocation clusters (see the area enclosed by the blue line in Figure 14) and dislocation rings (see the red circle in Figure 13), which is caused by defects such as interstitial atoms or vacancies caused by the proton radiation damage. The dislocation loops and linear dislocations observed in the literature [18,32,33] are basically the same. It is observed that after tempering the 5140 alloy steel at 480 °C and undergoing the effects of proton irradiation of different count fluences, the evolution process of its microstructure is shown in the dislocation loop and position. As the cumulative fluence of the proton irradiation increases, the density of wrong clusters also increases. This can be qualitatively characterized from the dislocation configuration and dislocation density shown in Figure 14a–c. When the movement of these defects is hindered or pinned by grain boundaries and precipitates, it promotes their proliferation or growth. Therefore, the density of dislocation rings or dislocation clusters caused by the irradiation will increase accordingly. This is consistent with the results reported in the literature [10,34,35,36,37].

In general, the mechanical properties of a material are directly related to its microstructure, that is, the smaller the grain size and the greater the dislocation density, the greater the strength of the material, but the elongation is reduced. In this paper, the proton irradiation reverses. The effect of the microstructure near the surface layer of the fire 5140 alloy steel is manifested in the increase in the density of dislocation rings and dislocation clusters, resulting in an increase in the yield strength and tensile strength of the tempered 5140 alloy steel, but the increase is only very small. This may be due to the 160 keV low-energy proton irradiation, the radiation damage formed only occurs in the very thin surface layer of the sample, so the macro mechanical properties of tempered 5140 alloy steel are deteriorated. This is mainly because the radiation damage caused by the proton radiation only acts on the very thin layer near the surface of the tempered steel so that it does not have a remarkable effect on the macroscopic mechanical properties [38]. Therefore, from the perspective of macro-mechanical properties, low-energy proton irradiation has little impact on the deterioration of the mechanical properties of tempered 5140 alloy steel. Consequently, it is concluded that this alloy can be applied in the exposed environment outside the space agency.

### 4.2. Proton Irradiation Hardening Mechanism of Tempered 5140 Alloy Steel

**Figure 15:**
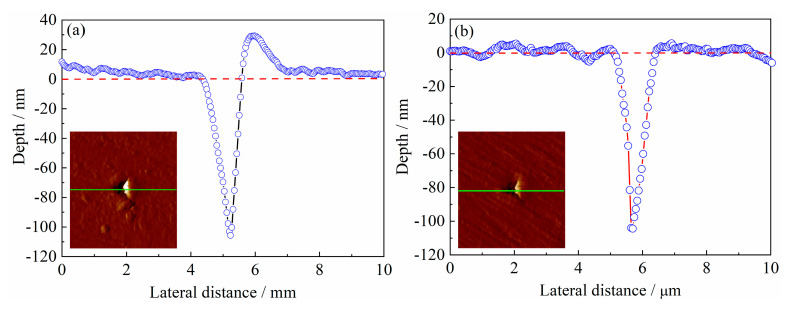
Nanoindentation profiles of tempered 5140 alloy steel before and after proton irradiation: (**a**) unirradiated, (**b**) after irradiation.

Figure 15 shows the nanoindentation before and after the proton irradiation. The deformed profile shows that before and after the proton irradiation, the dislocation density in the microstructure is different. Therefore, the mechanical properties of the material surface are also different. The damage caused by the proton irradiation forms crystal defects on the surface layer. These defects promote the movement and growth of dislocations and the segregation of solid solution atoms and the growth of precipitates. Due to the lack of traps formed by a large number of dislocations in the microstructure, the stress field around it attracts defects such as interstitial atoms that can move around and entangle with the surrounding dislocations to increase the nanohardness of the material [39]. In other words, the proton irradiation plays an important role in the surface irradiation hardening of the tempered 5140 alloy steel, resulting in an increase in the hardness of the surface layer. Therefore, the depth of its nanoindentation decreases as the cumulative fluence of the proton irradiation increases. This nanohardness value increases with the irradiation fluence.

Moreover, since the irradiation causes a series of matrix defects, the nanoindentation depth decreases. This causes a pinning effect on the dislocations, making the dislocation slip difficult. Therefore, radiation hardening is formed, and Figure 15 shows the effect of the radiation hardening. The nanoindentation outline shown in Figure 15a,b is the deformation curve marked with green lines in the illustration. When the tempered 5140 alloy steel is not proton irradiated, the deformation on both sides of the alloy nanoindentation pits is uneven. Figure 15a shows that a significant indentation bulge occurs on the right side of the pit. Moreover, the deformation on both sides of the nanoindentation pits of the tempered 5140 alloy steel after proton irradiation is relatively uniform (see Figure 15b), which may be attributed to the surface irradiation hardening caused by the proton irradiation [36,39].

## 5. Conclusions

In the present study, the effects of the proton irradiation with different cumulative fluences on the microstructure evolution, macro-mechanical properties, and nanoindentation mechanical properties of the tempered 5140 alloy steel are studied.

Tempering heat treatment and proton irradiation lead to changes in the microstructure and grain size of the 5140 alloy steel. It is found that as the tempering temperature increases, the size of the martensite lath increases. As the cumulative fluence of the proton irradiation increases, the apparent density of dislocations in the microstructure of the tempered 5140 alloy steel increases. The microstructure evolution mechanism shows that the density of dislocation rings or dislocation clusters caused by point defects such as vacancies and dissociated atoms formed by the proton irradiation damage increases accordingly, which leads to the formation of radiation hardening on the surface layer of the 5140 alloy steel.

The yield strength and tensile strength of the tempered 5140 alloy steel after proton irradiation increase as the proton irradiation cumulative fluence increases. However, the increase is very small and only in the range of 2.4–8.7% variety. Moreover, the change in elongation is reversed. This is because the irradiation damage caused by the proton irradiation only acts in the very thin layer near the tempered 5140 alloy steel surface. Therefore, it has no significant effect on the macroscopic mechanical properties.

The effect of the proton irradiation with different cumulative fluences on the nanoindentation hardness of the tempered 5140 alloy steel is evaluated by the nanoindentation technology. The nanoindentation hardness increases monotonously as the proton irradiation cumulative fluence increases. The hardening mechanism is due to a series of matrix defects caused by the irradiation, which causes a pinning effect on dislocations, making dislocation slipping difficult, and thereby forming irradiation hardening.

In the abovementioned comprehensive evaluation, the tempered 5140 alloy steel under the action of 160 keV low energy proton irradiation is investigated. It is concluded that the radiation damage formed on the ultra-thin surface layer of the sample causes alterations on the macro-mechanical properties and nanoindentation mechanical properties of tempered 5140 alloy steel. However, these changes do not have a significant impact on performance degradation, which can satisfy the service in the exposed environment outside the space agency cabin.

## Figures and Tables

**Table 1:** The chemical composition of the 5140 alloy steel (wt. %).

Materials	C	Mn	Si	S	P	Ni	Cr	Cu	Fe
5140 alloy steel	0.42	0.66	0.26	0.006	0.009	0.01	0.98	0.01	other

**Table 2:** Residual depth and nanoindentation hardness of tempered 5140 alloy steel before and after proton irradiation.

Pressure Load μN	Irradiation Dose Protons/cm^2^	Tempering Temperature								
		480 °C			520 °C			560 °C		
		E GPa	H GPa	D nm	E GPa	H GPa	D nm	E GPa	H GPa	D nm
4000	0	211.9	6.29	133.9	209.2	5.79	140.6	208.7	4.76	172.1
	2 × 10^14^	209.6	6.54	130.7	210.0	5.85	139.9	207.6	5.03	152.5
	2 × 10^15^	212.6	6.56	130.5	211.7	5.96	138.7	209.0	5.59	142.9
	2 × 10^16^	211.1	6.58	130.3	208.4	6.23	134.6	206.5	6.54	130.8
6000	0	205.8	5.87	176.9	203.9	5.41	185.5	202.1	5.04	192.4
	2 × 10^14^	206.1	6.12	172.8	205.2	5.68	180.5	203.1	5.38	186.1
	2 × 10^15^	206.1	6.45	167.4	206.3	5.93	175.9	202.2	5.61	181.6
	2 × 10^16^	207.4	6.72	163.6	205.2	6.23	170.1	203.2	6.15	172.3
8000	0	202.5	5.87	208.6	200.9	4.93	229.9	198.7	5.08	224.9
	2 × 10^14^	203.5	6.13	203.4	201.0	5.39	217.6	200.9	5.43	217.9
	2 × 10^15^	203.0	6.37	198.8	202.8	5.81	211.9	199.3	5.56	215.1
	2 × 10^16^	204.1	6.51	196.7	203.3	6.22	201.9	201.9	5.69	211.9
